# Exploring the Paradox of COVID-19 in Neurological Complications with Emphasis on Parkinson's and Alzheimer's Disease

**DOI:** 10.1155/2022/3012778

**Published:** 2022-08-31

**Authors:** Sachchida Nand Rai, Neeraj Tiwari, Payal Singh, Anurag Kumar Singh, Divya Mishra, Mohd. Imran, Snigdha Singh, Etrat Hooshmandi, Emanuel Vamanu, Santosh K. Singh, Mohan P. Singh

**Affiliations:** ^1^Centre of Biotechnology, University of Allahabad, Prayagraj, India; ^2^Faculty of Biosciences, Institute of Biosciences and Technology, Shri Ramswaroop Memorial University, Barabanki, India; ^3^MMV, Department of Zoology, Banaras Hindu University, Varanasi, India; ^4^Centre of Experimental Medicine & Surgery, Institute of Medical Sciences, Banaras Hindu University, Varanasi 221005, Uttar Pradesh, India; ^5^Centre of Bioinformatics, University of Allahabad, Prayagraj, India; ^6^Mahatma Gandhi Kashi Vidyapith, -221002, Varanasi, Uttar Pradesh, India; ^7^Clinical Neurology Research Center, Shiraz University of Medical Sciences, Shiraz, Iran; ^8^Faculty of Biotechnology, University of Agronomic Science and Veterinary Medicine, 59 Marasti Blvd, 1 District, 011464 Bucharest, Romania

## Abstract

Severe acute respiratory syndrome coronavirus 2 (SARS-CoV-2) is a human coronavirus (HCoV) that has created a pandemic situation worldwide as COVID-19. This virus can invade human cells via angiotensin-converting enzyme 2 (ACE2) receptor-based mechanisms, affecting the human respiratory tract. However, several reports of neurological symptoms suggest a neuroinvasive development of coronavirus. SARS-CoV-2 can damage the brain via several routes, along with direct neural cell infection with the coronavirus. The chronic inflammatory reactions surge the brain with proinflammatory elements, damaging the neural cells, causing brain ischemia associated with other health issues. SARS-CoV-2 exhibited neuropsychiatric and neurological manifestations, including cognitive impairment, depression, dizziness, delirium, and disturbed sleep. These symptoms show nervous tissue damage that enhances the occurrence of neurodegenerative disorders and aids dementia. SARS-CoV-2 has been seen in brain necropsy and isolated from the cerebrospinal fluid of COVID-19 patients. The associated inflammatory reaction in some COVID-19 patients has increased proinflammatory cytokines, which have been investigated as a prognostic factor. Therefore, the immunogenic changes observed in Parkinson's and Alzheimer's patients include their pathogenetic role. Inflammatory events have been an important pathophysiological feature of neurodegenerative diseases (NDs) such as Parkinson's and Alzheimer's. The neuroinflammation observed in AD has exacerbated the A*β* burden and tau hyperphosphorylation. The resident microglia and other immune cells are responsible for the enhanced burden of A*β* and subsequently mediate tau phosphorylation and ultimately disease progression. Similarly, neuroinflammation also plays a key role in the progression of PD. Several studies have demonstrated an interplay between neuroinflammation and pathogenic mechanisms of PD. The dynamic proinflammation stage guides the accumulation of *α*-synuclein and neurodegenerative progression. Besides, few viruses may have a role as stimulators and generate a cross-autoimmune response for *α*-synuclein. Hence, neurological complications in patients suffering from COVID-19 cannot be ruled out. In this review article, our primary focus is on discussing the neuroinvasive effect of the SARS-CoV-2 virus, its impact on the blood-brain barrier, and ultimately its impact on the people affected with neurodegenerative disorders such as Parkinson's and Alzheimer's.

## 1. Introduction

The SARS-CoV-2, coronavirus disease-2019 (COVID-19), pandemic has made the daily lives of people all across the world challenging with a significant family burden. Millions of people have lost their lives, and the number is increasing day by day [1]. Both economy and health have been seriously affected as a result of this pandemic [2, 3]. Severe acute respiratory syndrome coronavirus 2 (SARS-CoV-2) is the strain of coronavirus that has caused this COVID-19 pandemic [4–6]. This pandemic's global lockdown has resulted in severe psychological and mental stress among children and adolescents [7, 8]. Mental and psychological health is very important to maintain homeostasis in the central nervous system (CNS) [9]. This pandemic has led to a disturbance in the homeostasis of the CNS by affecting the cytokine concentrations and may be responsible for the initiation of many neurodegenerative diseases [10, 11]. The blood-brain barrier (BBB) is mainly responsible for preventing harmful substances from entering the CNS [12–14]. COVID-19 also causes harmful changes in the permeability of the BBB [15]. The spike in SARS-CoV-2 is mainly responsible for the alteration in the permeability of the human BBB, as suggested by 2D static and 3D microfluidic *in vitro* models [16]. Consequently, altered permeability and integrity cause the BBB to be more prone to developing different neurological and neuroinflammatory diseases in the CNS [13, 14]. Parkinson's disease (PD) and Alzheimer's disease (AD) are the two most common neurodegenerative diseases found all over the world that share almost similar mechanisms of progression [17, 18].

The research studies focusing on delineating the relationship between coronavirus disease and neurodegenerative disorders have provided conflicting results. Thus, a clear causal link could not be established. However, several mechanisms have been proposed by which COVID-19 might contribute to the development of PD and AD, such as the cytokine storm associated with severe COVID-19 which might trigger neuroinflammation and eventually lead to neurodegeneration. Several studies have found significantly high levels of inflammatory cytokines such as TNF-*α* and IL-6 in COVID-19 patients [19]. Similarly, earlier studies on Parkinson's disease have correlated high plasma IL-6 concentration with an increased risk of PD development [20]. Therefore, an increased possibility of neuroinflammation due to cytokine storm associated with COVID-19 cannot be ruled out.

The meta-analysis of the Parkinson's disease gut microbiome suggested alterations linked to intestinal inflammation [21]. As revealed by various studies, the development of PD is also linked to the gut microbiome and its dysbiosis [21, 22]. In a study, SARS-CoV-2 imbalanced the gut microbiome (dysbiosis) and intestinal inflammation indicated by elevated fecal calprotectin in COVID. Similarly, cognitive decline was observed during acute covid infection, but cognitive impairment was also observed post COVID [23, 24].

Medicinal plants like *Mucuna pruriens*, *Withania somnifera*, and *Tinospora cordifolia* and their active components like ursolic acid and chlorogenic acid, which are very beneficial for PD and AD, also show significant therapeutic activity in the management of COVID-19 patients. Recent data suggest that these plants and their bioactive components play a very crucial role in the clinical and preclinical studies for the management of COVID-19 patients [25–31]. Researchers are also working on the derivatives of the medicinal plants and their bioactive components to investigate their possible interaction among COVID-19 patients and in animal models. In this review, we present the viewpoint on the effect of COVID-19 on permeability of the blood-brain barrier. Then, we discussed the effect of this pandemic on PD and AD neurotropism, neuropathology, and neuroinflammation in a sequential manner. We have also discussed the infection and mortality rate among AD- and PD-related dementia.

## 2. Effect of COVID-19 on the Permeability of the Blood-Brain Barrier

The microvascular endothelial cells can form a blood-brain barrier and cover the central nervous system from heterogenic microorganisms and toxins present in the blood [32]. These cells express tight-junction proteins that prevent adjacent cell movement [33]. However, several identified viruses that can disrupt the blood-brain barrier are the Zika virus, West Nile virus, and the arbovirus [34, 35]. Several experimental procedures conducted using *in vivo* and *in vitro* BBB models have determined that these viruses can replicate in the brain's microvascular endothelial cells and stimulate tight-junction protein degradation and downregulation leading to BBB disruption [34, 36]. Likewise, Bleau et al. demonstrated the penetration ability of the coronaviruses in the CNS via the extreme hepatotropic mouse hepatitis type 3 virus and a weakly typed A59 hepatotropic hepatitis mouse. The infected mice type 3 have brain invasion related to increased BBB permeability.

The impact was linked with an induced level of zona occludens protein 1 expression, occluding, and VE-cadherin [37]. Considering the molecular relatedness of coronaviruses in their replication mode to infect the brain's microvascular endothelial cells, it was determined that other kinds of coronaviruses can also use similar activity pathways to do the same [38]. Significantly, CoV's existence has been recognized in the microvascular endothelial cells (brain) of the frontal lobe tissues, obtained from the postmortem examination of COVID-19 patients [39]. Also, a viral particle and CoV genomic content have been identified in the brain's neuronal cytoplasm, particularly in the region of the cortex and hypothalamus [40]. Evidence suggests that SARS-CoV-2 can cross the BBB accompanied by basement membrane disruption [41], and in another study, the S1 subunit of the spike protein of severe acute respiratory syndrome coronavirus 2 was found to cross the blood-brain barrier by adsorptive transcytosis and angiotensin-converting enzyme 2 was involved in its brain and lung uptake [42]. Hence, the infection via various respiratory viruses and SARS-CoV-2 may disturb the BBB integrity via distinct mechanisms. The viral particles can directly cause cellular stress associated with cytotoxic effects, which may lead to the disruption of the infected cell; for example, coronaviruses can stimulate cell apoptosis [43]. However, the activation of endothelial cells of the inflammatory reactions can increase protease expression, such as matrix metalloproteinase, promoting tight-junction protein degradation [44]. This evidence suggests that inflammatory responses probably play a significant role in stimulating BBB disruption.

The coronaviruses can be a source of BBB damage via the activation of inflammatory reactions linked with the dysregulation of these mechanisms [45]. Likewise, the stimulation of microvascular endothelial cells is associated with changes in the permeability of the BBB. For example, in the physiological state, the immune cell displacement into the CNS is precisely managed through processes conducted at the BBB stage [46]. Interestingly, the circulation of immune cells to the CNS is deficient and restricted to the particular immune subsets (innate and adaptive immune cell subsets), such as antigen-presenting cells, like dendritic cells, macrophages, and lymphocytes, which can manage the immune control in the CNS [47]. At the time of viral infection, the level of immune cell migration is increased. Therefore, it can be concluded that the enhanced CNS inflammatory reaction and systemic inflammation resulting from a viral infection may trigger the integrity of BBB and further progress into neurodegenerative disorders. These mechanisms may be supported via a histopathologic examination of the brain tissue in people with a SARS-CoV condition, where CD3^+^ T lymphocyte and CD68^+^ monocyte/macrophage pathological infiltration was found in the mesenchymal cells of the human brain [48].

Moreover, the process of infiltration is associated with the interaction of *β*1 and *β*2 integrins, which have an expression on leukocytes and their ligands, such as vascular cell adhesion molecule-1 (VCAM-1) and intercellular adhesion molecules (ICAM-1, ICAM-2) [49]. The ICAM and VCAM-1 are present on the microendothelial cell surfaces that stimulate the extravasation over the BBB; inflammatory responses have been cited. The activation and infection of microvascular endothelial cells via the typical neurotrophic viruses may increase the endothelial adhesion molecules [36]. This mechanism promotes viral infected immune cell trafficking to the CNS via a “Trojan horse” [50]. However, at the time of viral replication in the host cells, the impairment may be created due to SARS-CoV-2 (cytoplasmic virus), which stimulates the discharge of damage-associated molecular patterns (DAMPs) [51]. Damage-associated molecular patterns (DAMPs) are endogenous compounds released through impaired cells that interact with a pattern-recognition receptor (PRR), which stimulates the endothelial cells, neighboring epithelial cells like macrophages, and a high inflammatory condition. Once the viral-host cell interaction has been accomplished, the viral protein and genomes can also be identified through PPRs that induce an immune cell response [52].

Distinct PPRs can identify SARS-CoV-2, such as Toll-like receptors (TLRs), expressed in various cell lines along with macrophages, endothelial cells, and dendritic cells. The TLRs (TLR3, TLR7, TLR8, and TLR9) stimulate different activation pathways that provide the proinflammatory cytokines and other antiviral drug molecules in controlling the infection. Therefore, this reaction can be exacerbated and dysregulate cytokine production [53]. The other PPRs and NOD-like receptors (NLR) may induce the inflammasome complex and stimulate the activation state in a few cells: the epithelial, macrophage, and microvascular endothelial cells may lead to the overproduction of interleukin (IL)-1*β* and IL-18 [54]. However, these processes need to be examined in detail for novel coronaviruses. Evidence suggests that viral RNA can stimulate the typical melanoma differentiation-associated gene 5 (antiviral state) and retinoic acid-inducible gene 1, into which interferons (IFN) release IFN types I and III. Interferons are important molecules that help prevent viral infection by stopping viral replication [55].

COVID-19 patients having high IFN levels, mainly IFN I, have been recognized; these molecules avoid replicating viruses from the adjacent cells. They impact the viral infection, that is, the activation of interferon-stimulated gene expression, cytokine production, and induction of immunoreactive cells (monocyte, macrophages, and neutrophils) [56]. Likewise, the other coronaviruses may have a similar pattern of responses. Moreover, COVID-19 patients have an enhanced level of chemokines and cytokines, such as interleukin-1 receptor antagonist (IL-1RA), IL-2, IL-6, IL-7, IL-8, IL-9, IL-10, IFN-*γ*, TNF-*α*, and the granulocyte-macrophage colony-stimulating factor [57]. Interestingly, an increased level of IL-6 is associated with a poor prognosis for SARS-CoV-2 [58]. The overproduction and imbalanced amount of the existing molecules may be defined as a cytokine storm, significantly creating BBB disruption. The cytokine storm stimulates the induction of macrophages, neutrophils, monocytes, and platelets; few of these molecules may be associated with coagulation and complement systems and involved in pathogenic inflammatory responses. Few chemokines might attract some innate immune response cells: natural killer cells, monocytes, T cells, and dendritic cells. It activates the production of other kinds of cytokines, such as granulocyte colony-stimulating factor, monocyte chemotactic protein-1, macrophage inflammatory protein 1-*α*, monocyte chemotactic protein-1, and IL-10, which help in the recruitment of monocytes and lymphocytes and initialize the humoral cell response [59]. These processes can contribute to the severity of the neurological manifestation of COVID-19 infection in the BBB. SARS-CoV-2-infected patients with overinflammatory alteration may lead to excessive thrombin production that prevents fibrinolysis and induces complement pathways. This reaction leads to microthrombin deposition, thrombin inflammatory reaction, and microvascular dysfunction, related to the impaired BBB [60]. The undetermined processes that can aid the damage in the BBB are adaptive immune reactions. The antibody (Ab) generation across COVID-19 can react with a few brain microvascular endothelial cells and be disrupted via activation of the complement system (C3 and C4 proteins). Besides, the AB-based enrichment phenomenon may enhance the contribution and infection to the damage. This mechanism has been extensively used in Zika and Dengue viral infections, where the antibodies produced in the primary exposure cross-react with the second exposure and increase the rate of disease rather than neutralizing it [61].

### 2.1. COVID-19 and Parkinson's Disease

The current pandemic COVID-19 caused by severe acute respiratory syndrome coronavirus 2 (SARS-CoV-2) has resulted in a severe health crisis worldwide and led to a global virtual deadlock [62]. Even though significant preventive steps have been taken as a complete lockdown of economic and social life, social distancing has become a severe health problem for people with Parkinson's disease (PD) [63, 64]. Increased evidence determines the oxidative stress involvement that develops NF-*κ*B in COVID-19 [65].

The effect of SARS-CoV-2 infection on PD patients has been extensively discussed by several researchers worldwide. The potential impact of SARS-CoV-2 illness on PD patients is still unknown; thus, there is a shortage of scientific studies. Nevertheless, the pandemic situation has resulted in an emphasis on psychological factors. Considering the standard features, which show an essential role in PD, oxidative stress may worsen PD progression in COVID-19 patients and vice versa. Therefore, the pathophysiology of PD may put the individuals at a high risk of severe stress and lead to one of the several unseen miseries of the COVID-19 epidemic. The enhanced stress during a pandemic may have various adverse short- and long-term effects on PD individuals [66].

On the one hand, enhanced psychological stress can disrupt several motor functions, such as dyskinesias and tremors, if it decreases the potency of the dopaminergic drugs [67, 68]. On the other hand, enhanced stress may also reveal the potency of the hypokinetic rigid syndrome, probably through a decreased remunerative system [69]. It could lead to an improved PD diagnosis level throughout the epidemic as various animal trials reveal that prolonged exposure to severe stress may lead to loss of dopaminergic cells in response to a toxin [70–73]. Besides, this pandemic has resulted in reduced physical activities due to complete lockdown and social distancing; as a result, people have not been able to do physical work [74]. Current studies provide evidence that physical exercises may reduce the clinical features of PD progression [75, 76]. A more rigorous workout was linked with a better outcome than a moderately intense workout [77]. The fundamental mechanisms are still unknown, but it is beneficial to note that reduced physical activity during the COVID-19 epidemic may lead to worse motor symptoms in PD individuals. It has also contributed to enhanced psychological stress, another exasperating indication of PD [78]. Likewise, it also leads to severe non-motor issues such as constipation or insomnia, because of reduced physical activeness [79]. Simultaneous evidence suggests that the COVID-19 pandemic will severely impact individuals having neurodegenerative disorders as they have enhanced exposure to negative consequences of decreased physical activity and increased stress. Moreover, both melancholies can aggravate their motor as well as non-motor manifestations. These pandemic situations work as stressors aligned in time for such individuals [65, 80, 81]. It provides novel events for health professionals to analyze how the current pandemic directed PD progression in the present longitudinal discipline by taking biological biomarkers' assets. It also allows healthcare professionals to test which factors can protect the patients from the adverse impact of this catastrophe, by adding PD resilience. In essence, the current detrimental situations will also bring long-term positive consequences for several individuals with PD globally.

The SARS-CoV-2 infection causes significant concern for people who have already suffered from PD. Chaudhry et al. revealed the evidence of the impact of COVID-19 on PD patients [65]. They discovered that both COVID-19 infection and 6-hydroxydopamine (6-OHDA)-induced toxicity prompted caspase-2, 3, and 8 stimulation through the NF-*κ*B pathway culminating in the death of dopamine-containing neurons (dDCNs) [65]. Their findings prove that SARS-CoV-2 and PD might have common inflammatory pathways beneath the oxidative stress, as they have the essential indulgence of NF-*κ*B. Although it has a clear correlation with oxidative stress biomarkers and the chronicity of viral infections, such as hepatitis C, the SARS-CoV's clinical data is limited [82]. Therefore, in the presymptomatic situation, several facts suggest that surplus reactive oxygen species (ROS) and impoverished antioxidant systems play a significant role in the pathologic process of COVID-19 infection and lung infection progression [83].

The animal trial of the COVID-19 disease has shown an increased ROS level and disturbed antioxidant defensive mechanisms during the SARS-CoV-2 infection [38]. The healthcare professionals suggested that the onset of chronic lung injury in a COVID-19 condition may depend on the stimulating oxidative-stress machinery associated with innate immunity, which may induce transcription factor NF-*κ*B, resulting in aggravated proinflammatory host behavior [84, 85].

The coronavirus has been shown to spur caspase-based apoptosis required for the viral replication process [86]. According to various literature surveys, the PI3K/Akt signaling pathway induced via a collection of viruses helps in establishing a chronic infection, by delaying apoptosis in virus-infected cells, thus helping to complete the virus life cycle [87]. Accordingly, PI3K/Akt signaling may be a potential target against this COVID-19 pandemic and should be seriously considered. In addition, the role of oxidative stress, JNK, Akt, p38, and NF-*κ*B signal pathways has also been proven in influenza A virus (IAV)-induced acute lung injury; thus, these can also act as possible biomarkers in COVID-19-induced lung injuries [88]. The oxidative stress, such as IAV-mediated TLR4 and NF-*κ*B signaling pathways, may trigger SARS-CoV, increasing the host inflammatory reaction and leading to acute lung injury. Several studies have delineated the role of oxidative stress-mediated TLR4-TRIF-TRAF6 signaling pathways as an inflammatory response pathologic pathway that induces the implacability of chronic lung injury. The oxidative phospholipid is generated through the overproduction of lung macrophage-stimulated cytokines and leads to lung injury by TLR4-TRIF [89].

These oxidative phospholipids have also been recognized in animal and human lungs infected with the COVID-19 virus [90]. As is evident from these studies, a research focus should be kept on oxidative stress, as it might be the connecting link between the COVID-19 pandemic and neurodegenerative disorders. Similarly, in another study, upregulation of mitochondrial genes was also determined. These genes responded to oxidative stress, in the isolated peripheral blood mononuclear cells of recovered SARS-CoV patients [91]. Some of the genes included, such as *FOS*, *FTH1*, and *PRDX1*, have precise oxidative stress and provide remarkable ascent. The results suggest evidence of an association between inflammation, oxidative stress, and the pathogenic process of the COVID-19 infection. The virus infection has always been treated as a predisposing factor for developing PD and long-term neuron loss.

Several studies have shown the impact of dysfunctional mitochondria, mitochondrial gene upregulation, and the genes that respond to oxidative stress (an essential feature of neurodegenerative disorders) in cells recovered from COVID-19 patients. A COVID-19 study revealed the production of proinflammatory cytokines (CXCL-8, IL-6, CCL20, CCL3, CCL4, and IL-12) by dysfunctional mitochondria [41, 45, 92]. Among these proinflammatory cytokines, chemo-attractants, such as CXCL-8, promote neutrophil infiltration, thus contributing to ROS generation and protease activation that further contribute to damaged mitochondria [93].

Similarly, the consideration has kept on COVID-19 infection due to induction of a marked fundamental proinflammatory reaction. An eventual case-control analysis revealed that males with a high plasma IL-6 concentration have an increased risk of developing PD [94]. It is already known that SARS-CoV-2 infection occurs through TMPRSS2 and ACE2 [95]. Likewise, when compared with untreated rats, the TMPRSS2 expression was upregulated in rats treated with 6-hydroxydopamine, which is extensively used as a tool to model PD [96]. This examining procedure demonstrates that genes may codify, as these proteins are differentially regulated and crucial in disease development. Interestingly, in the past, coronaviruses have been associated with PD individuals. Significantly, intravenous antibodies for coronavirus types, MHV-A59, and MHV-JHM are increased in PD individuals, compared to people with other neurological disorders [97].

However, the influential role of viral infection in PD patients is still not known. Hence, a long investigating period of post-COVID-19 patients is required, but some characteristics of the chronic phase of this disease are very distinct. For example, a prior detection of gastrointestinal problems and anosmia and a high predominance of reduced recognition are identified in more chronic phases [98]. It is believed that the neurotropic pattern of the COVID virus is related to its activity, which produces respiratory symptoms, with approximately 89% of the people in intensive care units not being able to breathe voluntarily. Apparently, this is caused by a central dysfunction [99, 100]. However, gastrointestinal problems and hyposmia are the common non-motor symptoms in PD individuals during the prodromal stage; this is when neurodegeneration has been initiated [101]. Based on Braak's hypothesis, these indications suggest that it is the beginning phase of PD progression, which entails *α*-synuclein deposition in the anterior olfactory nucleus region and dorsal motor region. Most coronaviruses share similar viral anatomy, infection mechanism, and pathogenic processes. The viral penetration in the host cells is induced through dipeptidyl peptidase 4 (DPP4) and angiotensin-converting enzyme 2 (ACE2) [100]. Furthermore, human SARS-CoV-2 infection can cause chronic neurological difficulty with refractory status epilepticus, seizures, encephalomyelitis, encephalitis, leukoencephalopathy, cerebellitis, Guillain-Barré syndrome, and severe neuromyopathy. Evidence suggests that neurotropism and neuroinvasion are common manifestations of coronaviruses [102].

Like other coronaviruses, SARS-CoV-2 can infect cells via associating its spike proteins and the ACE2 receptor. For interacting like this, the spike protein must be cleaved through the transmembrane serine protease enzyme. The cells expressing both TMPRSS2 and ACE2 receptors are more affected by the SARS-CoV-2 infection [103]. Chen et al. revealed the expression of ACE2 by examining the information obtained from the brain transcriptome database. These receptors are overexpressed in SN and brain ventricles, scattered in excitatory and preventive neurons, and present in oligodendrocytes and astrocytes [104]. Besides, there is no strong evidence for the co-expressive mechanism of ACE2/TMPRSS2 in the brain region. Brann et al. revealed that the non-neurological sensory olfactory epithelium cells such as Bowman's gland cells, horizontal basal cells, and sustentacular cells express both TMPRSS2 and ACE2 receptors [105].

Hence, these cells may be the primary ones to be affected by SARS-CoV-2 infection. However, these non-neuronal cells can support the developed olfactory sensory neurons of the sensory epithelium. Supportive cells can be influenced by HCoV, sequentially transmitting the virus to the olfactory sensory neurons via axonal transport after those invading neurons in the olfactory bulb and then spreading to the CNS, where they cause inflammation [106]. The process of viral invasion by the olfactory bulb to the brain has already been reported to show a role in neurodegenerative disorder. It may trigger pathological aggregated protein transmission in a prion-like manner [107]. In the case of a retrospective study of COVID-19-infected individuals hospitalized in Wuhan, China, the researchers have found that out of 214 patients, 78 may have neurologic symptoms, defective memory, and cerebrovascular disorder occurrence in the more acute cases [98]. Dysgeusia and anosmia are also commonly reported in COVID-19 patients [108]. These investigations support the speculation that SARS-CoVs like coronaviruses can infect human brain cells.

### 2.2. Trigger for Future Neurodegeneration

**Figure 1:**
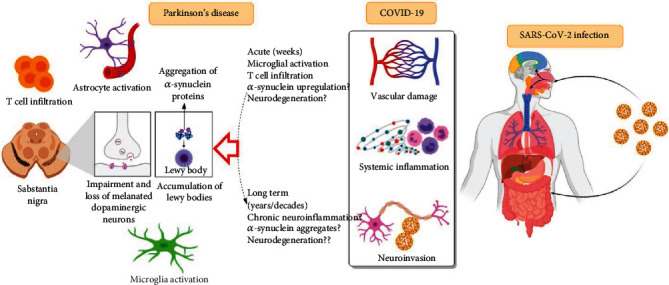
Schematic illustration of how COVID-19 infection impacts the pathology of Parkinson's disease. The COVID-19 infection might affect the brain in the following ways: through vascular damage, through systemic inflammation, and via direct neuroinvasion. These changes may lead to development of acute Parkinsonism due to microglial activation, T cell infiltration, and resulting in neurodegeneration due to *α*-synuclein upregulation. In addition to the development of acute Parkinsonism, COVID-19 may also elevate the PD risk in the long term due to sustained chronic inflammation and aggregation of *α*-synuclein.

The substantia nigra and cortex are the brain regions having a high risk of HCoV penetration through an ACE2 receptor. Often, this is the exact correlation in a neurodegenerative disorder and must not be taken as only a coincidence [39]. Lippi et al. have hypothesized the significant role of coronaviruses in the future development of neurological disorders, particularly in PD [109]. The author has hypothesized a new model for the neurodegenerative disease, as coronaviruses can promote *α*-synuclein accumulation, which is a significant element of Lewy bodies in the human brain, as has been suggested (Figure 1). The author focused on cellular pathways infected through the SARS-CoV-2 virus (proteostasis) to explain the phenomenon [109]. It is precise in providing efficient equilibrium and activates stress-induced mechanisms, which appear to be the same targets in neurodegeneration. Evidence has suggested that the infection of H1N1 of dopaminergic cells may result in the formation of *α*-synuclein aggregates and not of any other distinct protein recommended to focus on this mechanism positively. Therefore, *in vitro* models that determine the triggering changes in proteostasis may lead to the persistence of toxic impenetrable proteins [110]. These findings demonstrate that coronavirus infection may alter the PD neurodegeneration through progressive aging in brain cells or tissues (Figure 1).

### 2.3. The Impact of Coronavirus

SARS-CoV-2 has been recognized in brain autopsies and extracted from the cerebrospinal fluid of COVID-19-infected patients [111–113]. Currently, there are at least two well-developed facts about the association between neurodegenerative disorder and COVID-19, particularly for PD. On the one hand, there is a presence of antibodies against HCoV in the cerebrospinal fluid of patients who have already suffered from PD [97]. On the other hand, the second evidence suggests the penetration of the virus in the brain via the nasal cavity, which causes anosmia and hyposmia [114]. It has been reported that hyposmia is a common premotor characteristic of PD and the olfactory bulb is a chosen target for the deposition of *α*-synuclein pathogenicity [115]. The role of neurotropic virus pathogenicity in a neurodegenerative disorder can selectively target the basal ganglionic cells. Furthermore, few researchers also demonstrate that the longevity of the viruses in the nervous system infected cells for a longer time.

### 2.4. Modification of the Neurological Disease and Care Strategies

Donadio and coworkers state that the primary community depends on case-control analysis that describes the impact of symptomatic SARS-CoV infection on the motor or non-motor manifestation. In their group, 141 people who already had PD, in Lombardy, where there were 12 SARS-CoV-2-infected cases, had a mean age and course of disease duration similar to the controls [116]. The PD individuals may experience substantial damage to both motor and non-motor manifestations (mainly fatigue and urinary disorder) during the period of mild-to-tolerable COVID-19 infection, explicitly of aging and disease duration (Figure 1). Commonly, fatigue is reported as a dominant non-motor symptom during the COVID-19 infection in PD cases [114].

Comparatively, cognitive features were imperceptibly indulged; hence, none of them experienced autonomic damage. Medical disturbance can be observed in PD patients. It can be explained via infection-based mechanisms and damaged pharmacokinetics of dopaminergic treatment, requiring therapy adjustments in one-third of the cases [116]. The SARS-CoV-2 infection in PD patients cannot be limited to motor symptoms due to the incidental impact of the more prolonged exposure under lockdown, causing self-isolation, increased stress, and anxiety that should be considered. A current Iranian cross-sectional, case-control study evaluated the anxiety level among PD patients as compared to caretakers and familiar individuals. The occurrence of anxiety has been investigated in PD patient groups, who followed through with their caregivers [117]. Evidence suggests that the researchers have also demonstrated a strong association between severe anxiety in PD individuals and fear of having COVID-19 infection [117]. However, the other significant consequences of COVID-19 conditions are concerns about the considerable reduction in health activity.

The impact of reduced physical activity must not be neglected, as it may attenuate the progression of the medical symptoms in PD individuals. This evidence has been investigated by Shalash et al., who have determined the effects of COVID-19 on mental health, physical activeness, and quality of life in PD individuals. PD individuals harm their health care, cognitive ability, and interest in social life compared to PD controls [118]. Besides, Prasad et al. have demonstrated the implications and perceptions of SARS-CoV-2 infection in around 100 Indian PD individuals and their caretakers, which proved to have damaged the mental ability, physical wellness, and quality of life of these individuals [119]. This analysis may also shed light on the significance of managing these conditions preceding PD patients' care, specifically via taking telemedicine. It is not surprising to see PD individuals having impaired mental health during the lockdown. The immediate changes commonly need a flexible adjustment to new assets; these situations are strongly associated with the regular dopaminergic activity. Likewise, PD patients have experienced cognitive inability resulting in nigrostriatal dopaminergic depletion, which makes up the pathophysiologic substrate of PD [120]. To complicate this precarious situation of COVID-19 coexistence, there is less access to routine visits to hospitals to preserve PD patients from infection. Therefore, in the new era, taking care of patients has significantly changed, the most challenging concept for clinicians, as it has reinvented the work processes such as telemedicine, including digital visits, simple telephone consultations, and mail or text messages. As we advance in this direction, Goetz et al. accessed the validity and reliability of virtual-based MDS Unified Parkinson's Disease Rating Scale examination for comparing to official work. The E-Rehabilitation scheme can include virtual improvement programs as alternative strategies to deliver rehabilitation at a considerable level, which must be appreciated [121]. Although the telemedicine strategies are not well established universally, superior to the quality of regular in-person visits, evidence suggests that it associates with comparable results and can offer an excellent service and efficiency to PD patients. In short, the COVID-19 infection can complicate the PD clinical course, resulting from the severity of motor and non-motor manifestations, increasing stress and anxiety, and affecting the quality of life and mental wellness. In the era of COVID-19, telemedicine has played a significant role. Finally, various longitudinal and cross-sectional studies are required to find a causal association between clinical and severity of the SARS-CoV-2 infection, sequential inflammatory reactions having cytokine levels, and via identification in the CSF of PD individuals.

### 2.5. COVID-19 and Alzheimer's Disease

**Figure 2:**
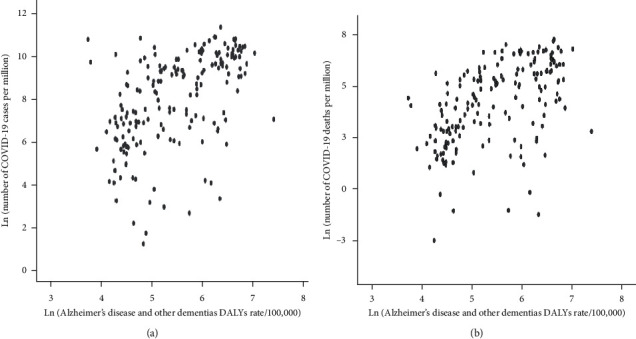
Scatter plot between Alzheimer's disease and other dementia disability-adjusted life year (DALY) rates. (a) The total number of COVID-19 cases per million. (b) The total number of COVID-19 deaths per million.

In our senior community, dementia is associated with a pandemic situation. However, the administration in the COVID-19 pandemic situation may also bring us more concerns [122]. The first concern is combining two potent risk factors, aging and dementia, for fatalities in COVID-19-infected patients. Likewise, the second concern is about the effect of associated coronavirus outbreak and dementia and the consequences of social distancing on the mental health of these weak patients, which needed a better prognosis [123].  Table 1 shows the global burden and mortality of Alzheimer's disease and other dementias, population, age, structure, and COVID-19. Results are classified according to (A) global burden of disease super-region classification and (B) World Bank income level-based classification. Similarly, Figure 2 exhibits a scatter plot between Alzheimer's disease and other dementia disability-adjusted life year (DALY) rates. It shows the total number of COVID-19 cases per million and the total number of COVID-19 deaths per million.

It is well understood that aged people are at high risk for fatalities after COVID-19 infection [124]. Very little data is available on older individuals infected with COVID-19, and limited reports have concentrated on above 80 individuals [125]. All of this has been demonstrated among older individuals having no dementia. Covino et al. provided results among dementia individuals having COVID-19 infection. Their results from this retrospect, single-centered, and observational examination in a Central Italy referral center for COVID-19 determine that the death risk does not depend on age [126].

In contrast, acute dementia itself might be a prominent risk factor among these individuals. Based on this evidence, Bianchetti et al. have determined the predominance, clinical representation, and results of individuals with dementia among those admitted in the hospitals with a COVID-19 condition [127]. Data obtained from 627 patients hospitalized in the Acute Hospital in Brescia province, Northern Italy, were examined retrospectively. Compared to people without dementia, patients with dementia have shown a high mortality rate of up to 40% [127]. Combining this investigation on dementia, mainly with the late onset of disease, may significantly risk fatality in corona patients. The neurological disease modification and care scheme are more vulnerable in AD patients due to neurological damage and a high neuropsychiatric symptomatology rate. This has been specifically proven at the stage of the compassionate outbreak that is SARS-CoV-2 infectious condition. Around 80% of AD individuals may show at least one or two neuropsychiatric manifestations throughout the infection. These factors are flexible and appear more progressive in AD, even if there may be early symptoms of prodromal stages. The neuropsychiatric manifestations of the AD medical spectrum include anxiety, depression, agitation, and emerging apathy, being exposed as frequent damages [128]. However, it is also considered being the effect of factors such as the enhanced rate of AD progression, altering meditational responses and reducing the patients' life quality. During the COVID-19 outbreak, for the first time, Boutoleau-Bretonniere et al. provided evidence of the impact of detention on the neuropsychiatric manifestation among AD individuals. The results suggest that only 30% of AD patients displayed neuropsychiatric alterations during the period of confinement. The confining period is significantly associated with their symptom severity and their caretaker's afflictions. The restrictions can worsen neuropsychiatric symptoms in AD patients, whereas no such manifestations were stimulated in people having more conserved cognition [129].

Other authors determined the severity of neuropsychiatric manifestations of agitation and impaired motor function, as the most affected indication among AD and MCI, during the complete lockdown during this COVID pandemic [130, 131]. The classic symptoms of AD patients with COVID-19 infection such as dyspnea, fever, and cough were less usual, while they specifically experienced drowsiness and diarrhea. Finally, that delirium can cause hypoxia, which is a prominent feature of COVID-19 infection and it can complicate the representation of dementia, although it is needed for dementia support and care [127]. However, these investigations can confirm the medical spectrum's anticipated risk, mainly the neuropsychiatric manifestations during the epidemic in AD individuals.

## 3. Trigger for Future Neurodegeneration

**Figure 3:**
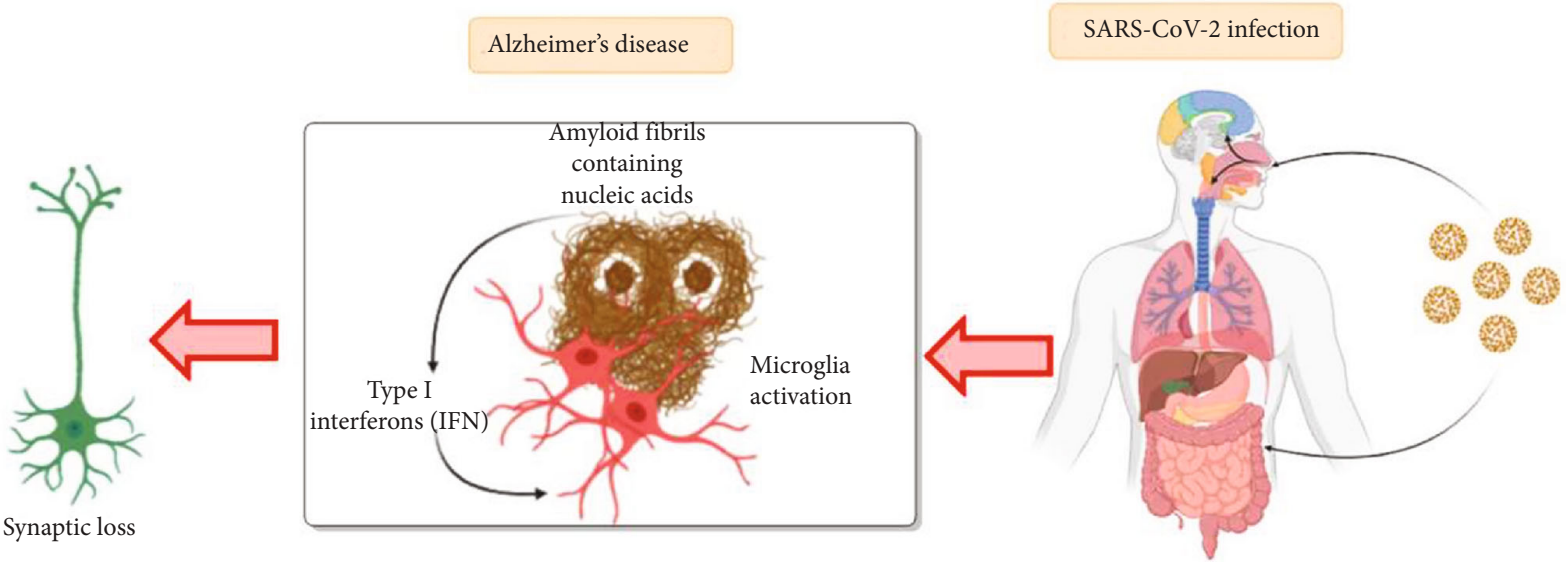
The potential interactions between Alzheimer's disease and SARS-CoV-2 infection. Synaptic loss takes place by inflammation mediated by type I interferon (IFN) after viral infection and in response to nucleic acid containing amyloid fibrils. The progression of IFN response occurs due to entrapment of viral particles by amyloid fibrils.

Despite having a long-term association between HCoV and its impact on the brain, it is not well understood; its efficient role in future neurodegeneration can be significant in AD research. Chronic results after the infection of COVID-19, often linked with the cytokine storm of distinct inflammatory responses, trigger enhanced proinflammatory cytokines (IL-1 and IL-6) [132]. In the case of AD patients, amyloid stimulates type I interferon (IFN) response; hence, it can create a perfect storm [133]. IFNs have an important role in AD pathology, thereby indicating nucleic acid containing amyloid fibrils stimulating the expression of genes responsible for IFN production. Microglia get activated by IFN, which gets associated with the nucleic acid-containing amyloid plaques, thus stimulating a proinflammatory response. IFN further activates complement cascade and leads to synapse degeneration (Figure 3).

It explains one of the questions that arise from presymptomatic individuals with undetected AD, who may see the stimulation of symptoms due to systemic inflammatory responses resulting from viral infection. Besides, several researchers have deliberated that infected people may have a high risk of impaired cognition after recovering from primary SARS-CoV-2 illness. The condition may directly negatively impact immune responses, and it also accelerates the preexisting cognitive deficiency and de novo stimulation of a neurodegenerative disorder. Based on the suggested evidence, it is possible to hypothesize that they may be at a high risk of generating neurodegenerative symptoms, which are unmasked via silent COVID-19 infection in the brain.

### 3.1. Management of Clinical Trial for the Alzheimer's and Parkinson's Patients Affected by COVID-19

The clinical features of COVID-infected patients are under great study. Globally, there have been 550 million confirmed cases of COVID-19, including 6.3 million deaths, reported as per WHO [134]. As of 11 July 2022, 12,130,881,147 vaccine doses have been administered. Therefore, it is crucial to scrutinize the risk condition of COVID-19-infected patients and their deaths. The lead factors affecting the enhanced transmission rate and medical severity remain unclear. The patients are suffering from other medical conditions such as cardiovascular disease, diabetes, and respiratory pathologies, and the elders especially are highly prone to the virus.

It is reported that age is a crucial risk factor for the SARS-CoVs-2 mortality rate. Age is the predominant feature of neurodegenerative diseases among older people. Thus, it is significant to analyze Alzheimer's disease and Parkinson's disease in SARS-CoV-2 infection [135]. SARS-CoV-2 is hazardous to older individuals with illness and neurodegenerative diseases like Parkinson's disease (PD) and Alzheimer's disease (AD). Currently, there is no proven evidence regarding the enhanced risk of COVID-19 for PD. Besides, severe PD symptoms associated with anxiety are often seen during the pandemic. PD patients, especially those who receive advanced therapy (brain stimulation or levodopa immersion), having a high fatality rate (40%–50%) [99]. In the case of patients with neurodegenerative disease, they are severely hampered by SARS-CoV-2 infection and require admission to the hospital (ICU) [136]. As previously mentioned, PD patients may have respiratory complications due to respiratory muscle bradykinin rigidity and dystonia, which can make life more challenging. Ingestion may also negatively impact these individuals, where saliva may pool in their mouth and cause breathing problems, which can complicate the management of COVID-19 patients. Even though no proven evidence supports the association of NDs with COVID-19 patients, it is examined worldwide as a future retrospective analysis concerning the clinical care strategy for ND patients admitted in the ICU. There are no current clinical guidelines. Therefore, an attempt should be made to ensure PD and AD patients continue to receive anti-PD and anti-AD therapy. For pneumonia patients on supporting previous PD treatment medicine, an equivalent levodopa dose is significant for avoiding muscle bradykinin rigidity and impaired breathing due to dopaminergic withdrawal. The apomorphine pump therapy and levodopa/carbidopa intestinal gel therapy on patients should be continued if it has already been implemented.

In some cases, PD medications should be adapted in hospitals, like severe kinetic individuals having dysplasia symptoms, where oral drug administration is no longer viable [136]. The easiest and cost-effective method to administer levodopa solution to patients is via a nasogastric tube. Initially, the apomorphic pump is not advisable to be used on patients in the ICU; however, they can be examined only if akinesia constitutes chronic effects. Likewise, another medicine that is trending is transdermal rotigotine, considerably less effective than apomorphine/levodopa. Until now, there is no strategic guideline to dictate an alternative therapy for ND patients in hospitals; it may be regulated using the clinical teams' experiences on a case-by-case basis [136].

### 3.2. Impact of Prolonged Lockdown due to COVID-19 in PD and AD Patients

**Figure 4:**
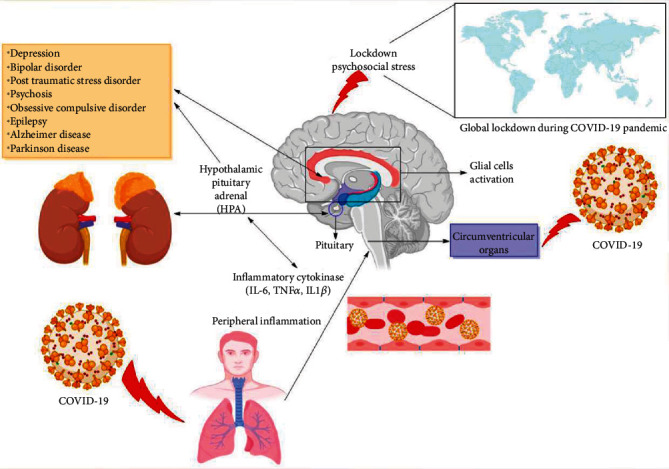
Schematic illustration of how global lockdown and SARS-CoV-2 lead to neuropsychiatric complications such as depression, anxiety, psychosis, and eventually leading towards neurodegenerative disorders. SARS-CoV-2 enters the body and causes systemic and tissue inflammation which compromises the blood-brain barrier (BBB). The combination of both psychosocial stress (caused by global lockdown) and disruption in the integrity of blood-brain barrier (BBB) floods the brain with an increased production and secretion of proinflammatory cytokines resulting in neuroinflammation.

The SARS-CoV-2 infection can drastically change the usual routine of PD patients through a lockdown and social distancing worldwide. Thus, many AD and PD patients experience a negative impact on their mental health due to prolonged lockdown (Figure 4). There are several reports which describe that the symptoms of AD and PD patients became worse and they have developed depressive and anxious symptoms, affecting their quality of life, compared to matched controls, during the epidemic. However, a detailed explanation is that the PD and AD pathophysiology naturally enhances the risk of severe depressive disorder, as decreasing dopamine levels reduce the stress-coping mechanisms. Currently, the data suggest that older individuals with concurrent medical problems (such as diabetes and hypertension) and patients having dementia are associated with an enhanced risk of COVID-19 and a high mortality rate. Thus, health-related interventions such as lockdown and social distancing have severely affected AD and PD patients' well-being (Figure 4). Social distancing is crucial for preventing COVID-19 infection and also in protecting at-risk populations such as older individuals with high mortality risk from SARS-CoV-2 condition [129]. Likewise, in India, elder individuals account for up to 104 million of the total population. The number of individuals living with Alzheimer's or dementia in India has been evaluated as 5.29 million (prediction for 2020) by the Alzheimer's and Related Disorders Society of India [137]. The effect of the epidemic on the symptoms of older individuals and patients with dementia is of grave concern to the healthcare professionals and dementia support organizations in the country. It is problematic, as stress has short- and long-term negative impacts on AD and PD patients. It has shown that cognitive stress can impair motor symptoms such as dyskinesia, tremor, and gait. Due to stress, the impact of the dopaminergic medications may also reduce, like the effect of levodopa on PD tremors [138].

It is significant to consider that physical workouts can impair PD progression and related stress. Moreover, social distancing during an epidemic significantly decreases mobility and physical activity, leading to a sedentary lifestyle. Accordingly, regular home-based exercise such as online classes for AD and PD patients is substantial for maintaining their overall health quality during COVID-19 [139].

### 3.3. Role of Aging-Related Neuroinflammation in Parkinson's and Alzheimer's Disease due to COVID-19 Pandemic

In human and animal models, SARS-CoV-2 can enter the brain along the brainstem precisely via the olfactory nerves and without any initial respiratory involvement [140]. The COVID-19 infection develops when the viral glycoprotein spikes get associated with ACE2 receptors. The ACE2 receptors are ubiquitously present in the human brain, not only in the medulla's cardiorespiratory centers but also in the striatum (dopamine neurons) [141]. However, two well-integrated pieces of evidence associate SARS-CoV-2 infection with movement disorder, particularly for PD. The first evidence, from over two decades ago, explains that antibodies exist against coronavirus in the CSF of PD patients [97]. Likewise, the second piece of evidence demonstrates the entrance of coronavirus into the brain via the nasal cavity, causing anosmia or hyposmia. It has been reported that hyposmia is a well-known premotor feature of PD. The olfactory bulb is the optimized target for alpha-synuclein pathologic deposition, which may be more than just fate [142].

### 3.4. The Relation between SARS-CoV-2 and Glial Cells in Parkinson's and Alzheimer's Disease

The glial cells (astrocyte and microglia) play a vital role in maintaining brain homeostasis and CNS response during neuropsychiatric and neurodegenerative disease conditions. These conditions often indicate a common neuroinflammatory need identified by the activated glial cells, releasing anti-inflammatory and proinflammatory cytokines, free radicals, chemokines, and neurotrophic factors. Evidence suggests that due to a large number of functions performed by the glial cells in the neuroinflammatory reactions, it is expected that the astrocytes and microglia have a significant effect on brain functioning during a viral infection, such as in COVID-19 patients. The extensive participation of glial cells in brain homeostasis management and viral infection expects that dysregulated glial cell function may explicitly and implicitly affect COVID-19 development. Aging is a crucial factor affecting the mortality rate in COVID-19 patients, even though the relation between SARS-CoV-2 and aging is still unclear. The age-based remodeling process is observed in astrocytes and microglia, which may ultimately cause damaged functional characteristics and contribute to the development of NDs (Figures 1 and 4) [142].

Similarly, the glial cells are present in various functional/morphological abnormalities in the older brain, such as increased ROS, decreased phagocytic activity, and mortality, proinflammatory cytokine production, and enhanced DNA mitochondrial impairment. These changes may result in the loss of the normal glial cell neuroprotective behavior and induce neuroprotective and neurodegenerative disorder; therefore, it is not seen in older COVID-19 patients. It is well known that impaired proinflammatory cytokine release may cause enhanced systemic symptoms and neurological damage in COVID-19-infected patients; however, whether glial cell activation is beneficial or harmful to the brain of COVID-19 patients is still a matter of examination.

## 4. Conclusion

The novel coronavirus infection has disrupted the world and its healthcare system unprecedentedly. This pandemic has also scared the viability and integrity of the present and near-future AD and PD research. However, local consequences will evolve and become distinct based on certain particular factors such as incidence, the linked death cases, the resource availability, and social changes that may control the COVID-19 pandemic. These local changes associated with AD and PD heterogeneity make simple generic suggestion inefficient. Therefore, the awareness about their probable consequences and alleviation approach adopted by the individual may prevent the harmful impact on patients with AD, PD, and their caretakers.

Moreover, further research is needed to demonstrate the detailed HCoV infection mechanism that leads to neuronal impairment, particularly if HCoV can truly exploit the associated pathways of CNS invasion. In that case, it may be quite interesting to analyze where the specific mechanisms combine with the specific neurological disease symptoms; this is yet to be resolved.

## Figures and Tables

**Table 1a:** (a) Global burden of disease study super regions

	COVID-19		Alzheimer's disease and other dementias		Population age structure		
Super regions	Cases per million (95% CI)	Deaths per million (95% CI)	DALY per 100,000	Death per 100,000	Age, median (IQR)	Population aged ≥65 years (%)	Population aged ≥70 years (%)
Central Europe, Eastern Europe, and Central Asia region	16866.33 (16613.94–17118.72)	287.84 (254.59–321.09)	501.02 (230.07–1078.94)	31.87 (7.76–85.40)	40.75 (36.65–43.15)	13.49	8.81
High-income region	24477.67 (24174.80–24780.54)	562.46 (515.99–608.93)	800.59 (372.40–1656.48)	59.25 (15.49–147.57)	41.60 (37.9–43.3)	18.31	12.24
Latin America and Caribbean region	18662.06 (18396.82–18927.31)	670.30 (619.57–721.03)	328.89 (140.60–717.62)	22.15 (5.59–56.73)	29.35 (26.9–32.85)	7.61	4.62
North Africa and Middle East region	7188.25 (7022.67–7353.82)	186.36 (159.60–213.11)	198.46 (87.40–439.09)	11.58 (2.83–30.52)	30.7 (23.2–32.4)	5.38	3.21
South Asia region	5810.05 (5661.09–5959.01)	84.72 (66.68–102.76)	145.00 (58.78–344.85)	8.76 (2.09–24.21)	26.25 (23.5–28.2)	5.65	3.27
Southeast Asia, East Asia, and Oceania region	570.42 (523.62–617.21)	14.82 (7.27–22.36)	359.62 (157.95–795.89)	19.86 (4.76–51.92)	29.3 (25.2–34.1)	9.21	5.19
Sub-Saharan Africa	1250.44 (1181.18–1319.71)	28.46 (18.00–38.92)	92.11 (37.71–210.29)	5.67 (1.41–15.43)	19.25 (17.9–21.75)	3.11	1.78

**Table 1b:** (b) World Bank classifications

	COVID-19		Alzheimer's disease and other dementias		Population age structure		
Income levels	Cases per million (95% CI)	Deaths per million (95% CI)	DALY per 100,000	Death per 100,000	Age, median (IQR)	Population aged ≥65 years (%)	Population aged ≥70 years (%)
High income	23552.65 (23255.42–23849.89)	512.73 (468.36–557.10)	771.05 (358.61–1596.43)	56.63 (14.78–141.36)	41.1 (36.2–43.2)	17.73	11.79
Upper middle income	6578.98 (6420.53–6737.44)	203.22 (175.29–231.16)	391.57 (173.18–854.67)	22.83 (5.63–59.56)	31.25 (28.8–38.35)	9.77	5.68
Lower middle income	4472.26 (4341.47–4603.04)	72.83 (56.11–89.56)	155.83 (64.15–361.11)	9.35 (2.28–25.53)	25.05 (20.35–28.4)	5.38	3.16
Low income	498.46 (454.71–542.21)	12.46 (5.53–19.36)	98.95 (41.17–227.55)	5.91 (1.47–16.09)	18.75 (17.5–19.4)	3.1	1.8

Global burden and mortality of Alzheimer's disease and other dementias, population age structure, and COVID-19. Results classified according to the (a) global burden of disease super-region classification and (b) World Bank income level based classification.

## Data Availability

No data were used to support this study.
